# Genome sequencing and comparative genomics provides insights on the evolutionary dynamics and pathogenic potential of different H-serotypes of Shiga toxin-producing *Escherichia coli* O104

**DOI:** 10.1186/s12866-015-0413-9

**Published:** 2015-04-03

**Authors:** Xianghe Yan, Pina M Fratamico, James L Bono, Gian Marco Baranzoni, Chin-Yi Chen

**Affiliations:** USDA, Agricultural Research Service, Eastern Regional Research Center, 600 E. Mermaid Lane, 19038 Wyndmoor, PA USA; USDA, Agricultural Research Service, Meat Animal Research Center, Clay Center, NE 68933 USA; U.S. Department of Agriculture, Eastern Regional Research Center, Agricultural Research Service, 600 East Mermaid Lane, 19038 Wyndmoor, PA USA

**Keywords:** STEC serogroup O104, Virulence, Plasmids, Genotyping, Comparative genomics, Next generation sequencing

## Abstract

**Background:**

Various H-serotypes of the Shiga toxin-producing *Escherichia coli* (STEC) O104, including H4, H7, H21, and H_¯_, have been associated with sporadic cases of illness and have caused food-borne outbreaks globally. In the U.S., STEC O104:H21 caused an outbreak associated with milk in 1994. However, there is little known on the evolutionary origins of STEC O104 strains, and how genotypic diversity contributes to pathogenic potential of various O104 H-antigen serotypes isolated from different ecological niches and/or geographical regions.

**Results:**

Two STEC O104:H21 (milk outbreak strain) and O104:H7 (cattle isolate) strains were shot-gun sequenced, and the genomes were closed. The intimin (*eae*) gene, involved in the attaching-effacing phenotype of diarrheagenic *E. coli*, was not found in either strain. Examining various O104 genome sequences, we found that two “complete” left and right end portions of the locus of enterocyte effacement (LEE) pathogenicity island were present in 13 O104 strains; however, the central portion of LEE was missing, where the *eae* gene is located. In O104:H4 strains, the missing central portion of the LEE locus was replaced by a pathogenicity island carrying the *aidA* (adhesin involved in diffuse adherence) gene and antibiotic resistance genes commonly carried on plasmids. Enteroaggregative *E. coli*-specific virulence genes and European outbreak O104:H4-specific *stx2*-encoding *Escherichia* P13374 or *Escherichia* TL-2011c bacteriophages were missing in some of the O104:H4 genome sequences available from public databases. Most of the genomic variations in the strains examined were due to the presence of different mobile genetic elements, including prophages and genomic island regions. The presence of plasmids carrying virulence-associated genes may play a role in the pathogenic potential of O104 strains.

**Conclusions:**

The two strains sequenced in this study (O104:H21 and O104:H7) are genetically more similar to each other than to the O104:H4 strains that caused an outbreak in Germany in 2011 and strains found in Central Africa. A hypothesis on strain evolution and pathogenic potential of various H-serotypes of *E. coli* O104 strains is proposed.

**Electronic supplementary material:**

The online version of this article (doi:10.1186/s12866-015-0413-9) contains supplementary material, which is available to authorized users.

## Background

The occurrence of a large outbreak involving enteroaggregative hemorrhagic *E. coli* (EAHEC) O104:H4 infections in Europe and North America in 2011 with prior reporting of similar strains in patients with severe infections and hemolytic uremic syndrome (HUS) in Asia, Norway, and the Republic of Georgia raises questions about the origin, evolution, genetic diversity, and virulence of these pathogens [1-7]. The German outbreak O104:H4 strain 2011-C-3493 had the characteristics of an enteroaggregative *E. coli* (EAEC); however, it carried the gene that encodes Shiga toxin 2a (*stx*_*2a*_), and thus can also be classified as a Shiga toxin-producing *E. coli* (STEC). Bielaszewska et al. [8] analyzed 80 isolates from this outbreak and found that they belonged to ST (sequence type) 678, similar to the HUSEC041 strain isolated from a patient with HUS in Germany in 2001. Moreover, the outbreak strains possessed a plasmid carrying CTX-M-15, encoding an extended-spectrum-β-lactamase, which is absent in HUSEC041. *E. coli* O104:H4 strains and O104 strains with other H-types showed genetic and phenotypic differences, including the presence of specific virulence and antibiotic resistance genes, the types of Shiga toxin genes, and their pulsed-field gel electrophoresis profiles [7,9,10].

Some of the most common flagellar H antigens associated with STEC strains include H_¯_, H2, H7, H8, H11, H12, H16, H19, H21, H25, and H28 [3,11]. Shiga toxin-producing strains belonging to serogroup O104 with the H7 or H21 flagellar antigens could be considered as more pathogenic [10]. According to the European Food Safety Authority, serogroup O104 has been reported from studies related to surveillance of animals and food by the EU Member States [12]. *E. coli* O104:H12 and O104:H21 were isolated from young cattle in Austria in 2009, O104:H7 from sheep and wild boar (O104/O127) in Spain, O104:H7 from sheep in India, from young cattle in Argentina, and from unspecified meat and sheep meat in New Zealand [13-19]. To date, other O104 serotypes attributed to human cases have been identified as O104:H21 [3], O104:H2 [20], and O104:H_¯_ [21]. In the U.S., there was an outbreak in Montana in 1994 caused by milk contaminated with STEC O104:H21 [22].

Differences in virulence and antibiotic resistance genes linked to disease severity and HUS among various O104 serotype strains, including O104:H11, O104:H21, O104:H7, O104:H2, O104:H12, and O104:H16 have been observed [10,23]. The differential phenotypic properties of O104 strains with H2, H4, H11, H12, and H21 antigens on selective media, such as Brilliance ESBL (extended-spectrum beta-lactamases), CHROMagar STEC, and CHROMagar O104 were also reported [24,25]. However, there is little information regarding the genetic basis of virulence of non-O104:H4 pathogens and how they might have emerged or were transmitted. Therefore, these serotypes should be fully investigated at the genomic level to identify genes important for virulence and for growth and survival in food. Moreover, besides EAEC-STEC O104:H4, STEC O104:H7, and STEC O104:H21, several *stx*-negative O104:H4 strains have been isolated from human patients in Central Africa [4,26,27] at different locations and at different times. Thus far, humans are considered as the only natural reservoir of EAEC strains [28], and the search for the source of the O104:H4 outbreak strain revealed that there was no animal reservoir for EAEC/EHEC/STEC O104:H4 isolates [29-32]. In contrast, STEC O104:H7 and STEC O104:H21 are reported to be associated with contaminated milk [22], feces of cattle, and/or from food of bovine origin [33]. Facilitated by next generation sequencing (NGS) technologies, the identification of mobile genetic elements, including plasmids, transposons, prophages, genomic islands, and chromosomal pathogenicity islands (PAIs) encoding virulence- and antibiotic resistance- associated genes, has provided insight into the genomic differences among environmental, animal, and clinical strains isolated from geographically dispersed areas. Unlike other STEC that cause serious human illness, STEC O104 strains lack the *eae* gene; however they can attach to intestinal cells. The EAHEC O104:H4 outbreak strain 2011C-3493 also lacked *eae*; however, this strain possessed a plasmid (pAA) that encodes for adherence fimbriae [9]. Studies found that not all O104 serotypes possess *aggR*, which encodes a regulator of virulence plasmid and chromosomal genes including aggregative adherence fimbriae and *aaiA-P* (*aggR*-activated island encoding a type VI secretion system), a characteristic of EAEC. EAHEC O104:H4 colonizes the human bowel through aggregative adherence fimbriae encoded by the EAEC plasmid (pAA). In the European O104:H4 outbreak strains, the intimin (*eae*) function was substituted by the plasmid-encoded aggregative adherence fimbrial colonization mechanism, and once attached, the cells were able to produce and deliver Shiga toxin 2a, resulting in severe illness and HUS. Recently, Beutin et al. [1] studied the possible uptake of the chromosome-encoded *stx*2-phage P13374 (from the German EAHEC O104:H4 strain) by EAEC strains of different serotypes. In this study, the authors concluded that the *stx*2-phage P13374 had a restricted host range, and its spread was dose-sensitive. One of the bovine-specific phages (P13803) was found to be capable of lysogenizing a *stx*-negative EAEC O104:H4 strain and converted it into an EAEC-STEC producing Stx_2a_ [1,34].

Previously published sequence data on O104:H4 isolates revealed variations in gene content among strains from different hosts and geographical sources [2,4,6,9,35]. Differences were observed in virulence- and resistance- associated genes among various H-serotypes of O104 strains. A better understanding of the genomic differences among strains belonging to different O104:H serotypes will help to reveal the basis for their pathogenicity and will provide information to understand how they evolved. The aim of this study was to sequence the genomes of the O104:H21 milk outbreak strain and a STEC O104:H7 cattle isolate, which had not been associated with human illness, and perform comparative analyses with the genomes of several *E. coli* O104:H4 strains isolated from different geographical locations. The resultant sequence analysis revealed notable differences among O104 strains belonging to H-serotypes H21, H7, and H4, including the presence of different plasmids and other mobile genetic elements.

## Results and discussion

### General genomic features of O104:H21 and O104:H7

The genomes of STEC O104:H21 strain 94–3024 that caused an outbreak of hemorrhagic colitis in Montana in 1994 linked to contaminated milk and strain O104:H7 RM9387, isolated from cattle feces, were sequenced. The genome size of O104:H21 strain 94–3024 was 4,902,583 bp, while that of O104:H7 RM9387 was 4,827,630 bp. O104:H7 RM9387 carried 4 plasmids, 3,173, 6,673, 6,819, and 169,634 bp in size; on the other hand, O104:H21 strain 94–3024 had only one large 161,447-bp plasmid. The G + C content for strains O104:H7 RM9387 and O104:H21 94–3024 were 50.8% and 50.7%, respectively, similar to that of other *E. coli* O104 strains. Information on all strains analyzed in this study, including genome size, GC content, and number of plasmids, is shown in (Additional file 1: Table S1).

### Comparison at the genomic level

In order to gain insight on the genomic differences among environmental, animal, and clinical strains isolated from geographically dispersed areas, the whole genome sequence of various EAEC/STEC O104 strains were analyzed. Whole-genome comparison showed that these 13 O104 strains shared a conserved core chromosomal backbone but contained various mobile genomic elements reflected in the differences in their genome sizes. Details will be further discussed in the next three sections.

**Figure 1:**
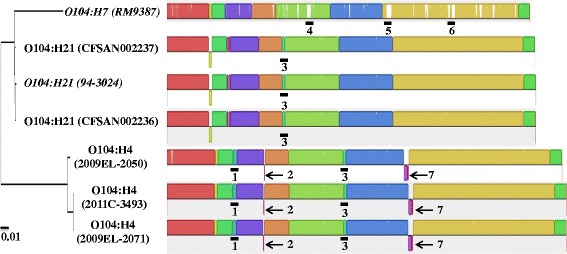
**Pairwise alignment of genomes from different H-types of O104 strains using MAUVE (default setting).** The right side represents the multiple genome alignment generated by Mauve software. Homologous blocks are drawn with same-colored blocks and internally free of genomic rearrangement (Locally Colinear Blocks or LCBs). Unique sequence regions to a particular genome were labelled with white color. Sequence regions below the center line indicate that the sequence blocks are in the reverse complement (inverse) orientation. The seven significant genomic dissimilarity regions are marked with black bold lines or arrows and numbered. The phylogenetic tree derived from the whole genome synteny alignment is shown on the left side of the Figure. The two strains sequenced in this study are italicized.

A comparison of seven O104 genomes, as visualized by the program MAUVE [36], including three O104:H21 strains isolated during an outbreak of hemorrhagic colitis in Montana in 1994 [37], is shown in Figure 1. Homologous regions that are conserved are shown in the same colors in Figure 1; gaps within a unique genomic region are in white. In this Figure, considerable conservation in the 7 genomes is revealed, although some serotype-specific regions were observed. For example, all three O104:H4 isolates possesed 3 prominent regions either completely missing (region #1, PAI carrying *aidA* and various type VI secretion-associated proteins; region #2, hypothetical proteins), or partially missing (region #7, tellurite resistance proteins and transporters) in O104:H7 and O104:H21 strains. Region #3 (phage_Yersin_413C; Additional file 2: Table S2) was present in all analyzed O104:H4 and O104:H21 strains, but missing in the sequenced O104:H7 strain. Other regions of significant genomic dissimilarity in O104:H7 include region #4 (various transporters and ATPase), #5 (transporters, etc.), and #6 (the adhesin gene, *iha*, Table 1).

**Table 1:** **Chromosome-encoded virulence genes, CRISPR, and IS elements profiling**

**Serotype**	**Strain name**	**Gene accession**	**Virulence, IS, and other biomarker genes**																		
			***fliCH4***	***fliCH7***	***fliCH21***	***wzxO104***	***wzyO104***	***stx2***	***tehB***	***iha***	***lpfa_O104***	***ent***	***tir***	***stx***_***1***_	***eae***	***Efa1***	***lpfa-O157***	***tccp***	***tccp2***	***IS629***	***CRISPR2***
O104:H7	RM9387	CP009104	-	+	-	+	+	+	+	-	+	-	-	-	-	-	-	-	-	-	-
O104:H21	94-3024	CP009106	-	-	+	+	+	+	+	+	+	-	-	-	-	-	-	-	-	-	-
O104:H21	BAA-178 (CDC 1994-3024) (CFSAN002236)	SRX319158	-	-	+	+	+	+	+	+	+	-	-	-	-	-	-	-	-	-	-
O104:H21	BAA-182 (CDC 1994-3023) (CFSAN002237)	SRX319159	-	-	+	+	+	+	+	+	+	-	-	-	-	-	-	-	-	-	-
O104:H4	2009EL-2050	NC_018650.1	+	-	-	+	+	+	+	+	+	-	-	-	-	-	-	-	-	+	+
O104:H4	2009EL-2071	NC_018661.1	+	-	-	+	+	+	+	+	+	-	-	-	-	-	-	-	-	+	+
O104:H4	2011C-3493	NC_018658.1	+	-	-	+	+	+	+	+	+	-	-	-	-	-	-	-	-	+	+
O104:H4	LB226692	SRR254209	+	-	-	+	+	+	+	+	+	-	-	-	-	-	-	-	-	+	+
O104:H4	TY-2482	SRX067313	+	-	-	+	+	+	+	+	+	-	-	-	-	-	-	-	-	+	+
O104:H4	C760-09	SRX078303	+	-	-	+	+	-	+	-	+	-	-	-	-	-	-	-	-	+	+
O104:H4	C777-09	SRX078304	+	-	-	+	+	+	+	+	+	-	-	-	-	-	-	-	-	+	+
O104:H4	C734-09	SRX078308	+	-	-	+	+	-	+	+	+	-	-	-	-	-	-	-	-	+	+
O104:H4	C754-09	SRX078302	+	-	-	+	+	-	+	+	+	-	-	-	-	-	-	-	-	+	+
O104:H^-^	4281-7	FM872416.1	na	na	na	na	na	na	na	na	na	na	na	na	+	na	na	na	na	na	na
O91:H21	B2F1	AGTI01000001-AGTI01000233	-	-	+	-	-	+	+	+	+	-	-	-	-	-	-	-	-	-	-
O113:H21	CL-3	NZ_AGTH01000001-NZ_AGTH01000258	-	-	+	-	-	+	+	+	-	-	-	-	-	-	-	+	+	-	-
O157:H7	Sakai	NC_002695	-	+	-	-	-	+	+	+	-	+	+	+	+	-	+	+	+	+	-
O145:H28	4865/96	AGTL01000001-AGTL01000345	-	-	-	-	-	+	+	+	-	+	+	-	+	+	+	+	+	+	-
O45:H2	03-EN-705	AGTK01000001-AGTK01000433	-	-	-	-	-	-	+	-	+	+	-	+	+	+	-	+	+	+	-
O26:H11	Str. 11368	NC_013361	-	-	-	-	-	-	+	+	+	+	-	+	+	+	-	-	-	+	-
O121:H19	MT#2	AGTJ01000001-AGTJ01000389	-	-	-	-	-	+	+	-	-	+	-	-	+	+	-	+	+	+	-
O103:H2	Str. 12009	NC_013353	-	-	-	-	-	+	+	-	+	+	-	+	+	+	-	-	-	+	-
O111:H-	11128	NC_013364	-	-	-	-	-	+	+	+	+	+	-	+	+	+	-	-	-	+	-
O111:H21	Str. 226	SRX160797	-	-	+	-	-	+	+	-	-	-	-	-	+	-	-	+	+	+	-

na: there is no whole genome sequence but partial fragment available.

**Figure 2:**
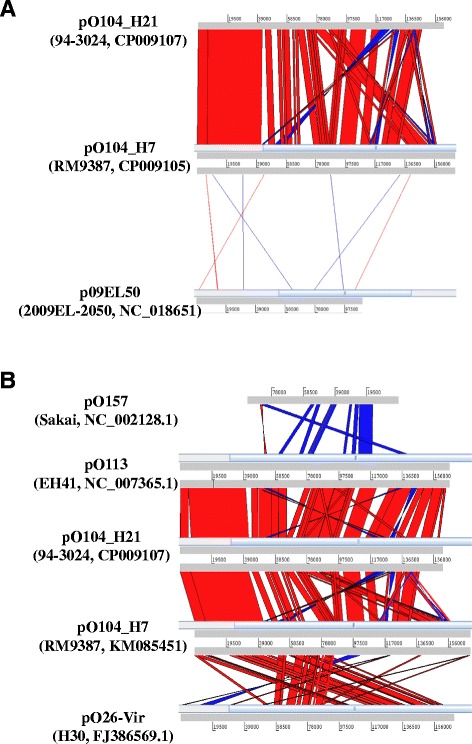
**Pairwise comparison of plasmids from different H-types of O104 isolates (A) and other STEC strains (B).** The plasmid sequences from various STEC strains were aligned and then visualized using the Artemis Comparison Tool (ACT). Plasmid sequence with similarity is shown by red (Homologous blocks) and blue lines (sequence block inversion) between the plasmids.

Each O104 strain carried 1–4 plasmids of different sizes, ranging from 3,173 to 169,634 bp (Table 2). Interestingly, the plasmids from the newly sequenced O104:H21 strain 94–3024, (161,447 kb, GenBank accession CP009107) and O104:H7 strain RM9387 (169,634 kb, GenBank accession CP009105) shared a high degree of overall sequence similarity to an STEC O113:H21 plasmid (strain EH41, GenBank accession NC_007365.1), less sequence similarity to plasmids from serotypes O157:H7 (NC_002128.1), and O26:H30 (FJ386569.1), and no sequence similarity to plasmids from the German outbreak O104:H4 strain (2009EL-2050, GenBank accession NC_018651.1, Figure 2A and B).

**Table 2:** **Comparison of large plasmids among STEC strains using BLASTN (coverage >90%, identity >90%)**

**Serotype**	**Strain name**	**Gene accession**	**Size (~Kb)**	**%GC**		**Virulence or other biomarker genes**														
					***AggR***	***AggB-C-D***	***saa***	***Ecf cluster***	***Hly cluster***	***excA***	***subA***	***SubB***	***espP***	***type IV pilus gene cluster***	***katP***	***espA***	***Etp cluster (type II)***	***msbB***	***stcE***	***toxB***
O104:H7	RM9387	CP009104	169,634	49.5	-	-	-	-	+	+	+	+	+	+	-	-	-	-	-	-
O104:H21	94-3024	CP009106	161,447	49.5	-	-	-	-	+	+	+	+	+	+	-	-	-	-	-	-
O104:H21	BAA-178 (CDC 1994-3024) (CFSAN002236)	SRX319158	na	na	-	-	-	-	+	+	+	+	+	+	-	-	-	-	-	-
O104:H21	BAA-182 (CDC 1994-3023) (CFSAN002237)	SRX319159	na	na	-	-	-	-	+	+	+	+	+	+	-	-	-	-	-	-
O104:H4	2009EL-2050	NC_018651	109,274	45.3	-	-	-	-	-	-	-	-	-	-	-	-	-	-	-	-
		NC_018654	74,213	47.1	+	+	-	-	-	-	-	-	-	-	-	-	-	-	-	-
O104:H4	2009EL-2071	NC_018662.1	75,573	47.2	+	+	-	-	-	-	-	-	-	-	-	-	-	-	-	-
O104:H4	2011C-3493	NC_018659	88,544	49.7	-	-	-	-	-	-	-	-	-	-	-	-	-	-	-	-
		NC_018666	474,217	47.1	+	+	-	-	-	-	-	-	-	-	-	-	-	-	-	-
O104:H4	LB226692	SRR254209	na	na	+	+	-	-	-	-	-	-	-	-	-	-	-	-	-	-
O104:H4	TY-2482	SRX067313	na	na	+	+	-	-	-	-	-	-	-	-	-	-	-	-	-	-
O104:H4	C760-09	SRX078303	na	na	-	+	-	-	-	-	-	-	-	-	-	-	-	-	-	-
O104:H4	C777-09	SRX078304	na	na	-	+	-	-	-	-	-	-	-	-	-	-	-	-	-	-
O104:H4	C734-09	SRX078308	na	na	-	+	-	-	-	-	-	-	-	-	-	-	-	-	-	-
O104:H4	C754-09	SRX078302	na	na	-	+	-	-	-	-	-	-	-	-	-	-	-	-	-	-
O157:H7	Sakai	NC_002128.1	92,721	47.6	-	-	-	+	+	-	-	-	+	-	+	-	+	+	+	+
O157:H-	3072/96	NC_009602.1	121,239	49.7	-	-	-	+	+	-	-	-	-	-	-	-	+	+	+	-
O113:H21	EH41	NC_007365.1	165,548	49.6	-	-	+	-	+	+	+	+	+	+	-	-	-	-	-	-
O26:H11	Str. 11368	NC_013369.1	85,167	47.5	-	-	-	+	+	-	-	-	-	-	+	-	-	+	-	+
	H30	FJ386569.1	168,100	50.2	-	-	-	+	+	+	-	-	+	+	+	-	-	+	-	+
O145	RM13514	CP006028	87,120	47.6	-	-	-	+	+	-	-	-	+	-	-	-	-	+	-	+
	RM13516	CP006263	98,066	49.7	-	-	-	+	+	-	-	-	-	-	-	-	+	+	-	-
	83-75	HM138194.1	90,103	47.5	-	-	-	+	+	-	-	-	+	-	+	-	-	+	-	+
O121:H19	MT#2	AGTJ01000001-AGTJ01000389	na	na	-	-	-	+	+	-	-	-	+	-	-	-	-	+	-	+
O103	APEC O103	NC_011964.1	124,705	50.8	-	-	-	-	-	-	-	-	-	-	-	-	-	-	-	-
	Str. 12009	NC_013354.1	75,546	49.1	-	-	-	+	+	-	-	-	-	-	-	-	+	+	-	-
O45:K1:H7	S88	CU928146	133,853	49.3	-	-	-	-	-	-	-	-	-	-	-	-	-	-	-	-
O111-aEPEC	D275	KC340959.1	115,452	53.1	-	-	-	-	-	+	-	-	-	+	-	-	-	-	-	-
O111:H21	Str. 226	SRX160797	na	na	+	-	-	-	-	+	-	-	-	+	-	-	-	-	-	-
O91:H21	B2F1	AGTI01000001-AGTI01000233	na	na	-	-	-	-	+	+	-	-	+	-	-	-	-	-	-	-

### Whole genome phylogenetic comparisons among various O104 H type strains and other LEE-negative STEC

**Figure 3:**
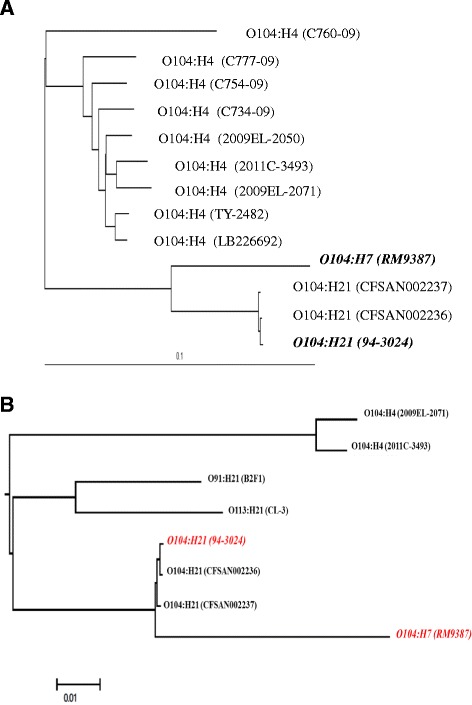
**Phylogenetic comparisons of** ***E. coli*** **O104 strains with various H-types from different sources (A) and to other LEE-negative STEC serotypes (B) by using whole genome sequences based on a MAUVE progressive alignment.**

In this study, we first inferred a whole-genome phylogeny of strains based on an alignment (Additional file 1: Table S1) based on an alignment of whole-genome contigs or complete full genome sequence mapped onto the complete closed genome sequence of the O104:H4 outbreak strain 2009EL-2050 (NC_018650.1) as the reference. A phylogenetic tree of O104 strains of different H-types from different sources revealed overall similar clustering of strains belonging to the H4 type, and that H7 and H21 had a closer relationship to each other than to O104:H4 (Figure 3A). The similarity of the profiles of the mobile genetic elements and the phylogenetic tree analysis indicated that O104:H7 and O104:H21 might share a similar evolutionary path since they are more closely related to each other than to O104:H4 strains. Phylogenetic comparisons among various LEE-negative STEC serotypes and different O104 strains showed that LEE-negative STEC serotypes O91:H21 and O113:H21 were genetically closer to O104:H21 and O104:H7 than to O104:H4 (Figure 3B). Our phylogenetic analyses (Figures 3) demonstrated that O104 strains with the same H-type clustered together (i.e. monophyletic) but their corresponding serogroups were cladded into multiple independent lineages (i.e. polyphyletic). Scattered distribution patterns of virulence factors and resistance genes, as well as phylogenetic analysis of these different O104 strains may suggest that strains from individual lineages might have acquired virulence genes and antibiotic resistance genes independently in parallel evolutionary processes.

### *stx* and *eae* genotypes

**Figure 4:**
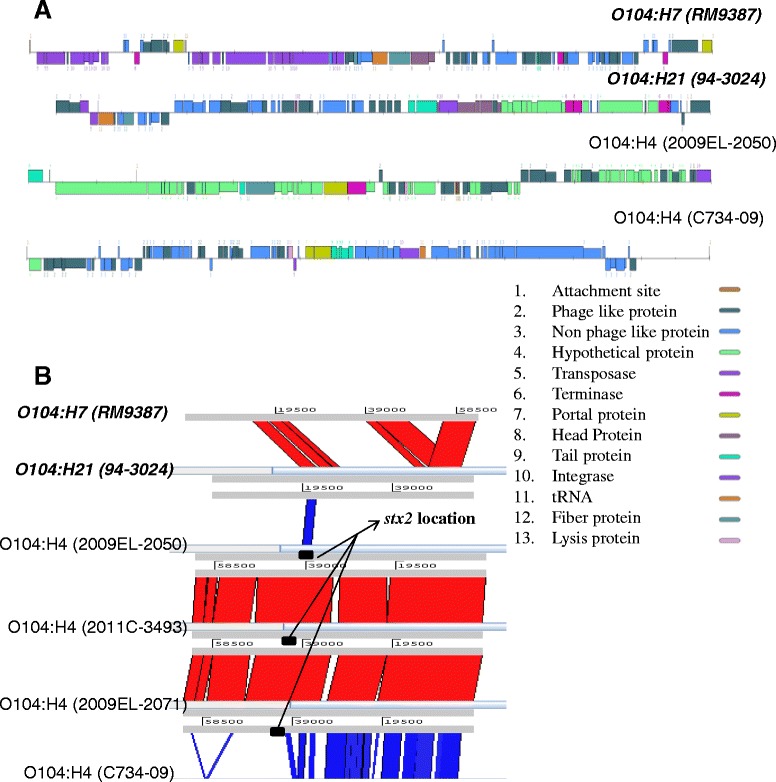
**Comparison of predicted** ***stx*** **prophage region in various H-types of O104 strains.** Top panel **(A)**: general gene features of the predicted *stx* prophage region among O104 strains; color and number codes for various function categories are below the main figure (Box). Bottom panel **(B)**: sequence alignment of the predicted *stx* prophage region visualized using ACT.

Shiga toxins Stx_1_ and Stx_2_ are recognized as the most important phage-encoded virulence factors in all disease-associated STEC strains, and the genes encoding for these toxins are located in the genome of mobile bacteriophages [38,39]. The *stx* phages are genetically distinct groups of temperate phages that can be identified in their prophage states inserted in the STEC chromosome, but also can be detected as phages released from the bacteria into the environment and/or animal hosts. *stx* phages can exist in polluted waters, feces, food, in humans, and the environment [40,41]. Generally speaking, phages could be used to detect and to derive lineage/evolution of newly emerged STEC strains from different hosts and/or environments [34,42-44]. The diagram in Figure 4A demonstrates significant diversity in gene sequence and structure to the predicted *stx* prophage region among serotypes O104:H7 (strain RM9387), O104:H21 (strain 94–3024), O104:H4 (strain 2009EL-2050, data not shown for other European outbreak strains), and O104:H4 (Africa strain C734-09, data not shown for other Africa strains). Sequence alignment and the linkage pattern analysis (Figure 4B) among O104 strains confirm that the region of predicted *stx* prophage shares weak sequence similarity among O104:H4, O104:H7, and O104:H21 strains. There was high sequence similarity among German outbreak strains (strain 2009EL-2050, 2011C-3493, 2009EL-2071) but not to Africa strain C734-09. There were at least four different types of *stx* bacteriophages found in the O104 strains analyzed in this study (Figure 4, Additional file 2: Table S2). The details of *stx* phage modular genetic structure in various O104 strains are illustrated on the diagram of Figure 4A. Genomic sequence analysis of the European outbreak O104:H4 strains showed that all isolates possessed *stx*_*2a*_ and some also carried *stx*_*2c*_, *stx*_*2d*_, or the combination of *stx*_*2a*_, *stx*_*2c*_, and *stx*_*2d*_ genes (data not shown). STEC strains that carry specific *stx*_*2*_ subtypes, especially *stx*_*2a*_*, stx*_*2c*_, and *stx*_*2d*_ tend to be more virulent [45]. In this study, activatable *stx*_*2d*_ was found to be the sole *stx* gene in our sequenced O104:H21 and O104:H7 strains. Sequence analysis also revealed that none of the O104 strains harbored the *stx*_*2e*_, *stx*_*2f*_, or *stx*_*2g*_ alleles.

*eae* is another important virulence gene of EPEC, EHEC, and STEC strains. The three prominent LEE integration sites selenocystyl-tRNA (*selC*), *pheU*, and *pheV* were previously described [46]. The different insertion sites of the LEE in various STEC suggest that the acquisition of LEE may depend on the strain genetic backgrounds, the environmental conditions to which the strain is exposed, and the mechanisms of genetic recombination. Our analysis showed that the O104:H7, O104:H21, and O104:H4 do not possess *eae* genes. It has been reported that serotype O104:H^−^ carried an *eae*-TAU (GenBank Accession: FM872416.1) variant. Strains expressing *eae-*TAU were generally restricted to and efficiently colonized Peyer’s patches in human intestinal mucosa [47]; so far, serotype O104:H^−^ has been found only in humans [21]. Schmidt and colleagues [48] reported a PAI in *E. coli* O91:H^−^ that occurs exclusively in a subgroup of STEC strains that are *eae* negative and contain the variant *stx*_*2d*_ gene, similar to O104:H7 and O104:H21 in this study.

**Figure 5:**
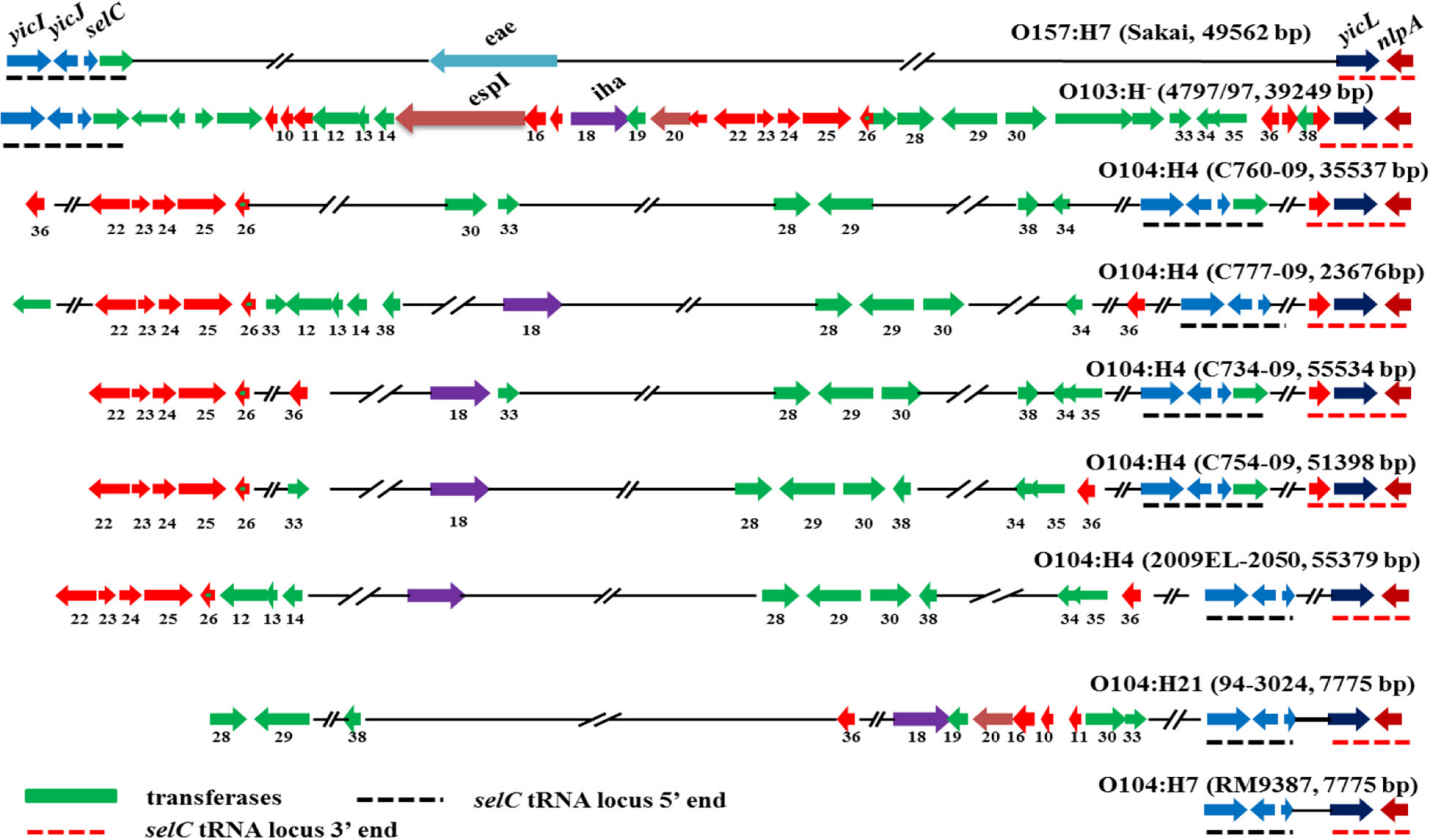
**Genomic feature comparison of the** ***selC*** **-tRNA loci among a variety of STEC serotypes.** Dashed lines (red and black color) represent the conserved gene regions. Sequence length between the black- and red-dashed lines (ranging from 7,775 to 49,562 bp) is indicated within the parenthesis. The ORFs are indicated by colors and numbers based on sequence similarity; arrow indicates the ORF orientation.

Also interestingly, a DNA fragment containing the LEE-encoded translocated-intimin receptor (*tir*) effector gene from serotype O104:H12 strain 4051–6 was sequenced (GenBank Accession# AB288103.1); however *tir* was missing in all O104:H4, O104H7, and O104:H21 strains examined in the current study (Table 1). The *tir* gene is typically encoded by the LEE, flanked by ORF19 (upstream) and *eae* (downstream) although the whole genome sequence of O104:H12 strain 4051–6 is not available currently. The functional Tir protein is translocated into the epithelial cell and is integrated into the cell membrane where it functions as a receptor for *eae*. Oswald and coworkers [49] showed that allelic differences in *eae* were associated with differences in tissue and host specificity. Thus, it is possible that O104 strains belonging to H7 or H21 serotype possess the EAEC or EHEC genetic background to acquire functional genes of various PAIs or *eae* variants (Figure 5, Additional file 3: Figure S1). A detailed description of Figure 5 is presented in next section.

### Identification and analysis of prophages, genomic islands, and/or pathogenicity islands

The number of predicted prophages varied greatly among various O104 H-type strains (Additional file 2: Table S2). Our analyses showed the distribution of different types of bacteriophages, particularly the *stx*_*2*_-carrying prophages, in the genomes of O104:H7, O104:H21, and O104:H4 supporting the hypothesis that the different H-serotypes of O104 may acquire diverse types of bacteriophages due to a possible genome adaptation to niches [50,51]. For instance, the similarity between bacteriophage types in the genomes of O104:H7 and O104:H21 shown in (Additional file 2: Table S2) suggested that they may come from the same evolutionary path or evolved in a similar environment.

An integrated comparative genomic program known as IslandViewer was used to identify genomic islands (GIs) and/or PAIs that may have been introduced into the genome by horizontal gene transfer (HGT). By directly applying IslandViewer for the identification of GIs in the genomes of O104:H21, O104:H7, and the O104:H4 reference strain (NC_018650), we found that the percentage of horizontally transferred genomic islands varied from 9.3%, 7.6%, and 12.3% among O104:H21, O104:H7, and the O104:H4 reference strain. The conserved GIs are identified by aligning all GIs in the reference strain O104:H4 to the GIs in other O104 strains. We found the conserved GI pools represented 70.9%, 65.7%, 65.5%, 49.1%, 44.3%, 19.3%, 15.3%, and 13.6% of the “reference genomic islands” from NC_018650 in O104:H4 strains LB226692, TY2482, C734-09, C754-09, C777-09, C760-09, O104:H21 94–3024, and O104:H7 RM9387, respectively. O104:H7 and O104:H21 appeared to be closer to each other and the O104:H4 C760-09 strain from Africa than to other O104:H4 strains, which is consistent with the phylogenetic tree in Figure 3A.

**Figure 6:**
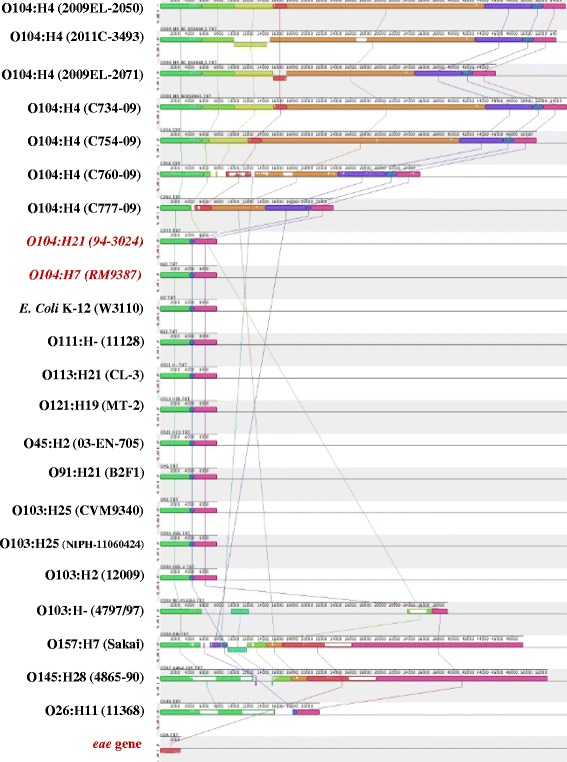
**Pairwise alignment of the** ***selC*** **-tRNA loci between** ***yicI*** **and** ***nlpA*** **among various** ***E. coli*** **strains using MAUVE.**

PAIs belong to a specialized class of GIs. They carry multiple virulence loci, may take up a large chromosomal region (>10 kb) of pathogens, are absent from non-pathogens, and usually have a different GC content from that of the core genome. A PAI from an O103:H^−^ strain which is inserted into the *selC* site between *yicI* and *nlpA* (shown in Figure 5) contains 40 ORFs encoding proteins with ≥ 50 deduced amino acids and one *selC* site (numbered sequentially from 1 to 41). The proteins encoded in this island include a novel serine protease EspI (ORF#15); an adherence-associated locus, similar to *iha* of *E. coli* O157:H7 (ORF#18); an *E. coli* vitamin B12 receptor (ORF #20); an *araC*-type regulatory module (ORF #22-26); and several other important and/or unknown function proteins. The remaining sequence consists largely of complete and incomplete insertion sequences, prophage sequences, and an intact phage integrase gene that is located directly downstream of the chromosomal *selC* [48]. A pairwise alignment of the *selC*-tRNA loci between *yicI* and *nlpA* to this typical O103 PAI shows some interesting genomic structural differences among O104 H-serotypes, O103:H^−^, O157:H7, non-O157 STEC, and other serotypes including *E. coli* K-12 (Figure 6). All of these strains shown in Figures 5 and 6 share the same gene structure at the N-terminal (*yicI*, *yicJ*, and *selC*) and C-terminal (*yicL* and *nlpA*), but none of these serotypes are genetically similar in the central portion of this typical PAI structure.

The genomic region of the *selC* site in O104:H7 and O104:H21 is 7775 bp and it encodes no known functional proteins. Here we propose the concept of “pseudo PAI”, a genomic region less than 10 kb in size that lacks key functional gene products typically found in O157:H7 and O103:H^−^ (Figure 5). Interestingly, strains carrying “pseudo PAI” in this *selC* site normally have smaller genome sizes and they could have lower pathogenicity if they also lack plasmids carrying virulence genes. It is important to note that the *eae* gene has at least 27 different variants and one of the subtypes, *eae*-TAU, has been isolated from patients with hemolytic-uremic syndrome in O104:H^−^ (Additional file 3: Figure S1) [52]. As shown in Figure 6, the presence and size of the PAI in the *selC* tRNA locus also shows no correlation with serotype. A “pseudo PAI” (7775 bp between genes *yicI* to *nlpA*) was found in strains of serotypes O104:H7, O104:H21, O111:H^−^, O113:H21, O121:H19, O91:H21, O103:H25, O103:H2, O26:H11, and even non-pathogenic *E. coli* K12. These differences in the size of the functional PAIs (serotypes O26, O145, O157, O104:H4, and strain 4797/97 O103:H^−^) or “pseudo PAI” among the same serogroup was also observed for O104 and O103:H^−^ . These observations are suggestive of the acquisition of the different PAIs by the same serotype, including O104:H4 strains due to their different ecological niches and/or the geographical regions where they were found. The *selC* site can be targeted by bacteriophage carrying LEE or LEE-like PAIs (e.g., O157, O26, etc.), bacteriophage harboring resistance genes (e.g. O104:H4) (shown in Figures 5 and 6) through horizontal genetic transfer (HGT) and adaptation to new ecological niches, or it can remain empty (e.g., O104:H7 and O104:H21).

As stated above, within different types of PAI inserted into the *selC* site, genes that may encode antibiotic resistance, are associated with altered metabolism, or that express virulence factors are located in the central portion of the island and are surrounded by mobility-associated genes (ORF with red color, Figure 5). Mobile genetic elements (ORF 28–30, 33, 34–36, and 38) are extensively interspersed throughout the genome of serotype O104:H4, but less so in O104:H21 (ORF 28, 29, 38, 19, 30, 33) and O104:H7 (no ORFs, except the pseudo PAI portion), particularly O104:H7 (Figure 5). However, Schmidt and coworkers [46] indicated that LEE in O91:H^−^ was flanked by prophage sequences and was devoid of IS-related sequences, which were only shown in O157:H7 in Figure 5. Our data may indicate that O104 H-serotypes could acquire PAI with or without an intact or a portion of LEE by phage transduction or HGT, which potentially could make O104 H-serotypes more pathogenic. At present, it is not known if the PAIs in different O104 H-serotypes were in the process of stepwise insertion of genes into the region, or stepwise deletion of genes from the “original” PAI, similar to those in O103 and O91:H- serotypes via complex mechanisms of horizontal transfer and/or recombination. A novel PAI, originally identified in O103:H^−^ and O91:H^−^, was also partially found in one O104 H^−^ serotype but showed some rearrangement (Additional file 3: Figure S1).

Based on our analyses, we propose an evolutionary model (Additional file 3: Figure S1) in the *selC*-tRNA site of various H-serotypes of *E. coli* O104 strains in a stepwise insertion fashion. This model suggests that O104:H^−^ and O104:H4 may derive from the ancestral O104:H7 or O104:H21 by the acquisition of antibiotic resistance genes (O104:H4) or *eae*-TAU carried by a prophage/PAI (O104:H^−^) and other genetic components, such as the plasmid carrying *aggR* gene. Various O104 strains were also shown to carry *stx*_*2*_ [53] (Table 2), implicating that within the O104 serogroup there are multiple acquisition events of prophage, GI, and/or PAI. PAIs can excise at different frequencies depending on growth conditions [54-56]. This suggests that genetic versatility is needed for the survival of *E. coli* in diverse environments, which may be relevant regarding its host specificity, as well.

### Chromosome- and/or plasmid- encoded virulence genes, CRISPR, and IS elements among O104 strains

The most noticeable difference in the genomes of these O104 serogroups was the presence of various types of mobile genetic elements, and the variations in the numbers of chromosome-encoded virulence genes. The H7, H21, and H4-specific *fliC, wzx, wzy, eae, bfpA* (plasmid-based), *stx*_*1*_*, stx*_*2*_*, efa1, ent/espL2, tir, nleB* and *nleE* (OI-122 specific), *espK* (prophage CP-933 N), *tehB, iha*, and O104- and O157-specific *lpfa, tccp, tccp2, IS629* integrase, and the CRISPR2 sequence were used to define serogroup and pathogroups of O104:H7, O104:H21, and other O104:H4 strains. Based on the genotypic differences, *E. coli* pathotypes can be categorized as atypical EPEC (*eae* only), typical EPEC (*eae* and *bfpA*), STEC (*stx*_*1*_ and/or *stx*_*2*_ with or without *eae*), EHEC (*eae* and *stx*_*1*_ and/or *stx*_*2*_), *aggR*-positive EAHEC (absence of *eae*, but with *stx*_*1*_ and/or *stx*_*2*_ and *aggR*), and *aggR*-negative EAHEC/STEC (absence of *eae* and *aggR* but with *stx*_*1*_ and/or *stx*_*2*_). These virulence factors and O- and H-antigen specific genes are useful for molecular characterization of EPEC, STEC, EHEC and EAHEC strains at the genomic level (Table 1). The *E. coli* O104:H4 European outbreak strain contains a *stx*-encoding phage similar to 933 W with only one nucleotide polymorphism in each of the subunits (*stx*_*2a*_ and *stx*_*2b*_) compared to 933 W. The German outbreak O104:H4 strains (Additional file 1: Table S1, see the origin of strain) also contain genes encoding several important serine protease autotransporter toxins, including *sepA* (*Shigella* extracellular protein A), *sigA* (*Shigella* IgA protease-like homologue), *pic* (protein involved in intestinal colonization), *aap* (dispersin), *aatPABCD* (dispersin transporter), and others; however, none of these were found in the sequenced O104:H7 and O104:H21 strains (data not shown). Aside from C760-09, O104:H4 strains from Africa did not carry noteworthy virulence factors other than the *iha* gene; however, C777-09 possessed the *stx*_2_ gene, thus indicating potential virulence (Table 1). Strain O104:H7 RM9387 and all O104:H21 isolates carried virulence genes that included *lpf* (long polar fimbriae) and *iha* (IrgA homologue adhesin) that are associated with EHEC and colonization of the gut, but lacked the *aggR*, *aggB-C-D* modular genes (Table 2).

The *stx*_*1*_*-* and *stx*_*2c*_*-* prophages are normally flanked by integrative element IS*629* at both ends in almost all O157 and non-O157 STEC, including O26, O45, O111, O121, and O145. Our analysis of the *stx* prophages indicated that the transposable insertion sequence element IS*629* may be a driver of *stx*_*1*_ and *stx*_*2c*_ prophage evolution among O104:H7, O104:H21, and other LEE-negative STEC strains. We have hypothesized that *stx* genes may excise from African strains after infection, owing to a functional change of a putative integrase near the *yqgA* (inner membrane protein) gene due to frameshift- and nonsense-mutations. After transposition, there is only one IS*629* left in O104:H4. IS*629* insertion is highly biased toward prophages and prophage-like integrative elements. Both IS*629* and IS*602* are not present in O104:H7 and O104:H21 but exist in all O104:H4 strains (Table 1). IS*602* also appears to be part of kanamycin (and related aminoglycosides) resistance transposons [57]. In addition, O104:H4-specific CRISPR2 was not found in any of the other STEC strains examined in this study (Table 1), including O104:H7 and O104:H21. The presence and the functions of CRISPR2 [58] may play a significant role in O104:H4 for maintaining a high degree of genomic plasticity and flexibility for adapting to host and environmental changes. Analysis of IS*629* and CRISPR distribution among O104 strains might be useful for identification and detection of specific O104 strains and population genetic analysis, as well as molecular epidemiology studies. The presence of IS*629*, CRISPR, and prophage regions (data not shown) may potentially contribute to the larger genome size of O104:H4 (Additional file 1: Table S1 and Figure 1).

There was no apparent correlation between serotypes and plasmid profiles among O104:H4, O104:H7, and O104:H21 strains, as well as other non-O104 STEC strains (Table 2, Figure 2). There was marked variability in the numbers and types of genes carried by the plasmids in the O104 strains (Tables 2 and 3); none of the 13 *E. coli* O104 isolates carried the same set of plasmids. STEC O104:H7 RM9387 had four plasmids, while most other O104 strains carried only 1 or 2 plasmids. The following genes used to define EAEC, STEC, and EHEC strains were located on some plasmids: *aggR*, *aggBCD* cluster (encoding major fimbrial subunits), *saa*, *ecf* cluster, *ehx* cluster, *etp* cluster (type II secretion system apparatus), *toxB* (putative adhesion), *katP* (catalase-peroxidase), *espP, espA, sepA* (serine protease sepA precursor)*, subAB*, typeIV pilus gene cluster, *mabB*, *stcE* (zinc metalloprotease), and *excA* (exclusion-determining protein), in agreement with other previous reports [10,59-62]. These genes are useful for the molecular detection and characterization of STEC, EAEC, and EHEC strains. A comparison of the various O104 strains revealed that *subAB* (a potent toxin involved in inducing cell death), the *ehx* cluster, *espP*, and *excA* were found in O104:H7, O104:H21, and LEE-negative O113:H21 strains, but not in O104:H4 strains. Therefore, it is suggested that virulence of the O104:H21 outbreak strain may be due in part to plasmid-associated virulence genes, including *subAB*, the *ehx* cluster, and *espP* (Table 2). In Table 2, only O104:H4 strains and an O111:H21 strain carried the transcriptional activator *aggR* and/or the aggregate *B-C-D* gene cluster (Table 2). *subAB* was more prevalent in LEE-negative than in LEE-positive STEC strains and likely contributes to the progression to severe disease [63], since the O113:H21 strain was involved in an outbreak with cases of hemolytic uremic syndrome (Table 2).

**Table 3:** **Profiling of antibiotic resistance genes in O104 and other STEC strains**

**Serotype**	**Strain name**	**Chromosome- and/or plasmid-encoded antibiotic resistance genes**																			
		***mdtL***	***vancomycin***	***beta-lactamase***	***emrE***	***ksgA***	***emrB/QacA***	***mdtG***	***mdaB***	***ydeB***	***dacC***	***folA-O157***	gentamicin resistance protein*	streptomycin 3’-kinase*	tetA (class A) *	tetA (class D) *	***folA***	beta-lactamase2*	aph*	erythromycin resistance repressor*	floR*
O104:H7	RM9387	+	+	-	+	+	+	+	+	+	+	+	-	-	-	-	-	-	-	-	-
O104:H21	94-3024	+	+	-	-	+	+	+	+	+	+	+	-	-	-	-	-	-	-	-	-
O104:H21	BAA-178 (CDC 1994-3024) (CFSAN002236)	+	+	-	-	+	+	+	+	+	+	+	-	-	-	-	-	-	-	-	-
O104:H21	BAA-182 (CDC 1994-3023) (CFSAN002237)	+	+	-	-	+	+	+	+	+	+	+	-	-	-	-	-	-	-	-	-
O104:H4	2009EL-2050	+	+	+	+	+	+	+	+	+	+	+	-	+	+	-	-	-	-	-	-
O104:H4	2009EL-2071	+	+	+	+	+	+	+	+	+	+	+	-	+	-	-	-	-	-	-	-
O104:H4	2011C-3493	+	+	+	+	+	+	+	+	+	+	+	-	+	+	-	-	+	-	-	-
O104:H4	LB226692	+	+	+	+	+	+	+	+	+	+	+	-	+	+	-	-	+	-	-	-
O104:H4	TY-2482	+	+	+	+	+	+	+	+	+	+	+	-	+	+	-	-	+	-	-	-
O104:H4	C760-09	+	+	+	-	+	+	+	+	+	+	-	-	+	-	-	-	-	-	-	-
O104:H4	C777-09	+	+	+	-	-	+	+	-	-	+	+	-	+	-	-	-	-	-	-	-
O104:H4	C734-09	+	+	+	-	+	+	+	-	+	+	-	-	+	+	-	-	-	-	-	-
O104:H4	C754-09	-	-	+	-	-	+	+	-	-	-	-	-	+	-	-	-	-	-	-	-
O157:H7	Sakai	+	+	-	-	+	+	+	+	+	+	+	-	-	-	-	-	-	-	-	-
O145:H28	4865/96	+	+	-	-	+	+	+	+	+	+	+	-	-	-	-	-	-	-	-	-
O45:H2	03-EN-705	+	+	-	-	+	+	+	+	+	+	+	-	-	-	-	-	-	-	-	-
O113:H21	CL-3	+	+	-	-	+	+	+	+	+	+	+	-	-	-	-	-	-	-	-	-
O26:H11	Str. 11368	+	+	-	-	+	+	+	+	+	+	+	-	-	-	-	-	-	-	-	-
O121:H19	MT#2	+	+	-	-	+	+	+	+	+	+	+	-	-	-	-	-	-	-	-	-
O103:H2	Str. 12009	+	+	-	-	+	+	+	+	+	+	+	-	-	-	-	-	-	-	-	-
O111:H-	11128	+	+	+	+	+	+	+	+	+	+	+	-	+	-	-	-	-	-	-	-
O111:H21	Str. 226	+	+	-	-	+	-	+	+	+	+	+	-	-	-	-	-	-	-	-	-
O91:H21	B2F1	+	+	-	+	+	-	+	+	+	+	+	-	-	-	-	-	-	-	-	-

*: plasmid-encoded.

### Antibiotic resistance gene profiling

There were a number of genes related to antibiotic resistance in O104:H4. The O104:H4 outbreak strains were resistant to at least 14 different antibiotics including ampicillin, sulfonamide, cefotaxime, ceftazidime, streptomycin, sulfamethoxazole, trimethoprim, cotrimoxazole, tetracycline, and nalidixic acid, but were susceptible to imipenem, kanamycin, gentamicin, chloramphenicol, and ciprofloxacin [62]. The O104:H4 outbreak strain carried a plasmid, pESBL, that encodes for extended spectrum *β*-lactamase CTX-M-15 [64,65]. Although the actual plasmid sequences were not available, genomic analysis showed that the four O104:H4 strains isolated from Africa carried some resistance genes (both chromosomal and plasmid-borne), but not as many as the O104:H4 strains of European origin (Table 3). On the other hand, O104:H7 and O104:H21 strains did not carry the plasmid-borne antibiotic resistance genes found in the German outbreak O104:H4 strains (Table 3). These antibiotic resistance genes listed in Table 3 may have been acquired through horizontal gene transfer by transposon-based plasmid integration (Table 3, Additional file 3: Figure S1). The analysis of the plasmid profiles of various O104 H-types and other STEC serotypes (Table 2) is consistent with the observation that EHEC plasmid-encoded genes (Table 2, GenBank accession CP009105 and CP009107), including *hly* cluster, *excA*, *subA/B*, *espP*, were only found in STEC O104:H7 and O104:H21 strains, but not in O104:H4 strains [10].

## Conclusions

The milk-associated outbreak STEC O104:H21 strain 94–3024 and the O104:H7 cattle isolate RM9387 were shot-gun sequenced, analyzed, and compared to elucidate the potential relationship, origin, and evolution of bacterial pathogenesis in various O104 H-serotypes. The observed variation between different O104 H-serotype genomes demonstrated that genome-wide divergence likely occurred via acquisition and loss of genomic islands, prophages, and plasmids. The genetic diversity in O104:H7, O104:H21, and O104:H4 serotypes was reflected in differences in virulence and resistance genes carried on the chromosome and on plasmids, suggesting their independent evolution demonstrated by different distribution and types of genetic mobile elements.

Genetic diversity of *stx*_*2*_ bacteriophages among O104:H4, O104:H7, and O104:H21 is illustrated in Figure 4. Further studies are needed to define the sources (cattle and other animals, fresh produce, the environment, humans) of these *stx* phages and to determine the frequency of lysogenization of *E. coli* O104:H7 and O104:H21 and phage origin by comparing to *stx*_*2*_-carrying phages from *E. coli* O104:H4 and other non-O104 EAEC/STEC strains. Their roles in dynamic bacterial genome evolution have been increasingly highlighted by the fact that many sequenced bacterial genomes contain multiple prophages carrying a wide range of genes, such as *stx* and antimicrobial resistance genes.

Bacteriophages are major genetic factors promoting horizontal gene transfer (HGT) between bacteria. In this study, a “pseudo *selC*-tRNA site PAI” was defined containing perfect direct repeats and transposons, but was less than 10-kb in length (Figure 5, Additional file 3: Figure S1). The results in Figure 5 indicated that O104:H4 and O104:H21 may represent various insertion intermediates of the O104:H7 strain generated in the course of O104 evolution. Our data provide new insights into the potential activities of the functional prophages embedded in bacterial genomes and may lead to the formulation of a novel concept of inter-prophage interactions in prophage communities. That is to say, strains containing prophage without genetic defects may be potentially more capable of spreading (gaining or losing) important virulence determinants such as *eae* and other genetic traits to other bacterial strains (Figure 6). Our findings suggest that more research is needed to understand the potential roles of prophage in HGT between bacteria and in the evolution of bacterial pathogens.

A key virulence factor, *stx*_*2*_, was found in all European O104:H4 outbreak strains, but only in one of the strains of African origin (C777-09) (Table 1). This finding supports the contention that similarly virulent O104:H4 isolates could be widespread; however, they could be genetically different due to their adaption to specific niches. It is also suggestive that both O104:H7 and O104:H21 may have evolved into pathogenic STEC strains due to the acquisition of *stx,* other virulence genes, and antibiotic resistance genes such as *mdtL, emrE, ksgA, ydeB, dacC, folA-O157*, and others from other STEC. It is reasonable to suggest that genetic variation of STEC O104 may partly be due to adaptation to local environments and interactions with other bacteria and hosts. Serotypes O104:H^−^ and O104:H12 have been isolated from humans and associated with HUS, thus it is also important that future studies examine the pathogenic potential of different O104 H-types, other than H4, H7, and H21.

## Methods

### Strains, genome sequencing, assembly, and annotation

STEC O104:H21 strain 94–3024 and O104:H7 strain RM9387 were obtained from the Centers for Disease Control and Prevention (Atlanta, GA) and from Dr. Robert Mandrell at the USDA Agricultural Research Service, Western Regional Research Center (Albany, CA), respectively. Ion Torrent libraries were prepared following the manufacturer’s recommended library construction procedures. Ion Torrent 316-chips with the 200-bp OneTouch kit was used for the generation of sequencing data on the Ion Torrent Personal Genome Machine (PGM). High molecular weight DNA for PacBio sequencing was extracted using Qiagen Genomic-tip 100/G columns and a modified manufacturer’s protocol as previously described [66]. Ten micrograms of DNA was sheared to a targeted size of 20 kb using a g-TUBE (Corvaris, Woburn, MA.) and concentrated using 0.45X volume of AMPure PB magnetic beads (Pacific Biosciences, Menlo Park, CA) following the manufacturer’s protocol. Sequencing libraries were created using 5 micrograms of sheared, concentrated DNA and the PacBio DNA Template Prep Kit 2.0 (3Kb - 10Kb) according to the manufacturer’s protocol. The library was bound with polymerase P5 followed by sequencing on a Pacific BioSciences (PacBio) RS II sequencing platform (with chemistry C3 and the 120-min data collection protocol). A fastq file was generated from the PacBio reads using SMRTanalysis Version 2.1 and error-corrected reads were created using pacBioToCA with self-correction [67]. The longest 20X of the corrected reads were assembled with Celera Assembler 7.0 [65]. The resulting contigs were polished using Quiver [68] and annotated using a local instance of Do-It-Yourself Annotator (DIYA) [69] for initial gene prediction and frame shift verification. The annotated genome sequence was imported into Geneious (Biomatters LTD., Auckland, New Zealand) and duplicated sequence removed from the 5′ and 3′ ends to generate the circularized chromosome. The chromosome was reoriented with the *dnaA* gene at the 5′ end. Reoriented circular chromosomes were reanalyzed using Quiver and a final annotated chromosome was generated with DIYA. Hybrid error correction and *de novo* assembly of single-molecule sequencing reads were evaluated and confirmed by Ion Torrent sequencing. The complete genomes of O104:H21 and O104:H7 used in this study were submitted to Prokka [70] to predict genes and annotate all assemblies, followed by manual checking for the final genome submission to NCBI. The identification of potential frame shifts and pseudo-genes of these two query finished genome sequences were also identified using the NCBI online service Microbial Genome Submission Check [71].

### Genome comparisons

The Artemis comparison tool, ACT version 10 [72] was used to plot nucleotide similarities (blastn) between O104:H serotypes and other non-O104 STEC. The comparison of particular pathogenicity/genomic islands, plasmids, and prophages was performed using the ACT alignment program at the default settings. These predicted genomic islands and prophages were identified from sequence alignments and breakpoint sites and were further manually curated. The gene name and locus ID were directly assigned based on the NCBI Reference Sequence files.

Mauve was used for comparing and visualizing a number of genomes. By applying O104:H4 strain 2009EL-2050 (NC_018650.1) genome as the “reference” strain, all FDA draft raw sequences for O104:H21 (Table 2, strains BAA-178 and BAA-182) downloaded from SRA (http://trace.ncbi.nlm.nih.gov/Traces/sra/sra.cgi?view=search_obj) and our two newly sequenced genomes were re-assembled and ordered based on reference genome. In general, progressive Mauve was applied for whole genome comparison. Homologs and/or orthologous relationship of the nucleotide sequences for profiling of virulence- and resistance-associated genes between genomes were determined using NCBI Basic Local Alignment Search Tool (BLAST) with the following criteria: identity >80%, e-value <1e − 10, and coverage >90%. The identified homologs and/or orthologous genes were further manually curated and confirmed.

### Phage identification and analysis

Prophages and putative phage-like elements in the O104:H4 reference strain 2009EL-2050 (NC_018650.1) and the newly sequenced O104:H7 and O104:H21 genomes were analyzed using prophage-predicting PHAST Web server [73]. Regions identified algorithmically as “intact”, “questionable”, and “incomplete” by PHAST, and regions sharing a high degree of sequence similarity and conserved synteny with predicted “intact” prophages, were marked as prophages. The extent of sequence similarity and synteny among these predicted prophage sequences were then aligned and presented using software ACT.

### Identification and comparison of pathogenicity islands and other genomic islands

IslandViewer [74] was used to predict genomic islands and/or pathogenicity islands (PAIs) [75] in the O104:H4 reference strain 2009EL-2050. The predicted genomic islands and/or pathogenicity islands were then used as the “reference genomic islands template” for the identification of corresponding genomic islands from various H serotypes of O104 isolates and other non-O104 STEC genomes based on 90% identity and 90% coverage (90%, 50 nts). The LEE protein sequences from the top 7 STEC serogroups (O26, O45, O103, O111, O121, O145, and O157) were used as query sequences for large-scale BLAST score ration analysis and BLASTP to detect the presence of LEE islands and its associated genes (homologs). The LEE pathogenicity island insertion site was determined using BLASTN to identify the contig and alignment coordinates of the intimin gene, *eae*, among all O104:H serotype strains listed in this study. The presence of the LEE carrying *eae* and its flanking genes was confirmed by manual inspection of the annotation for this region, and/or by detection of the coordinates of genes encoded within the LEE of O157:H7 strain EDL933.

### Identification and comparison of virulence factors and antibiotic resistance genes

Only bacterial genes experimentally confirmed to be involved in bacterial pathogenesis and antibiotic resistance were collected through an extensive literature mining in PubMed. This was followed by reference mapping to confirm their existence with corresponding raw sequencing reads. SNP discovery for RpoB (mutations in *rpoB*, β-subunit of RNA polymerase, can result in antibiotic resistance) was also performed (data not shown).

### Phylogeny tree construction

The phylogenetic relationship of the two sequenced O104:H7 and O104:H21 strains and other O104 strains was visualized and constructed using MAUVE whole-genome alignment and presented with the program Mega6 based on features in the genome or specific regions such as PAIs (Figure 6).

### GenBank accession numbers

The complete genome and plasmid sequences of O104:H21 94–3024 strain and O104:H7 RM9387 strain were deposited in NCBI GenBank database under the accession numbers: CP009104 (O104:H7 RM9387 chromosome), CP009105, KM085451, KM085452, KM085453 (O104:H7 plasmids pO104_H7, pO104_H7_s1; pO104_H7_s2, pO104_H7_s3), CP009106 (O104:H21 94–3024 chromosome) and CP009107 (O104:H21 plasmid pO104_H21).

## Additional files

Additional file 1: Table S1. Strain background and general gene/genome information of the 14 different O104 strains used in this study. Additional file 2: Table S2. Distribution and general information of the predicted prophages in various H types of O104 strains. Additional file 3: Figure S1. Proposed evolutionary model of the selC-tRNA site from various H-types of *E. coli* O104 strains.

## Abbreviations

LEE: Locus of enterocyte effacement
STEC: Shiga toxin-producing *Escherichia coli*
*aidA*: Adhesin involved in diffuse adherence
EAHEC: Enteroaggregative hemorrhagic *Escherichia coli*
HUS: Hemolytic uremic syndrome
PAIs: Pathogenicity islands
