# Supplementary material for: Genome sequencing and comparative genomics provides insights on the evolutionary dynamics and pathogenic potential of different H-serotypes of Shiga toxin-producing Escherichia coli O104
Source: BMC Microbiol. 2015 Apr 3;15:83. doi: 10.1186/s12866-015-0413-9 (PMC4393859; doi:10.1186/s12866-015-0413-9)
Supplement: Additional file 1: Table S1. — Strain background and general gene/genome information of the 14 different O104 strains used in this study. [file 12866_2015_413_MOESM1_ESM.docx]

**Table S1**. Strain background and general gene/genome information of the 14 different O104 strains used in this study

| **Serotype** | **Strain name** | **Accession # (GenBank)** | **Size of Chromosome (bp)** | **No. of Plasmids** | **G+C (%)** | **# of Genes** | **Sequencing Technology** | **Strain information** |
| --- | --- | --- | --- | --- | --- | --- | --- | --- |
| O104:H- | 4281-7 | FM872416.1 (*eae* gene) | na | na | na | na | na | aEPEC strain (Brazil, 2009) |
| O104:H7 | RM9387 | CP009104 | 4,827,630 | 4 | 50.8 | 4,505 | A combination of PacBio SMRT™ and Ion Torrent | Cattle (US) |
| O104:H21 | 94-3024 | CP009106 | 4.902585 | 1 | 50.7 | 4,539 | A combination of PacBio SMRT™ and Ion Torrent | Human stool from an outbreak, Montana, US, 1994 |
| O104:H21 | BAA-178  (CDC 1994-3024)  (CFSAN002236) | SRX319158 | 4,898,581 | 1 | 50.7 | na | Ion Torrent | Human stool from an outbreak, Montana, US, 1994 |
| O104:H21 | BAA-182  (CDC 1994-3023)  (CFSAN002237) | SRX319159 | 4,897,294 | 1 | 50.7 | na | Ion Torrent | Human stool from an outbreak, Montana, US, 1994 |
| O104:H4 | 2009EL-2050 | NC_018650.1 | 5,253,138 | 2 | 50.7 | 4,969 | a combination of Illumina and 454 technologies | *stx_2a_*+; *aggr+, aatA+,* and positive for fermentation of sorbitol, lactose  and β-glucuronidase.  eae; ehxA-, and *CefS* (Republic of  Georgia) |
| O104:H4 | 2009EL-2071 | NC_018661.1 | 5,312,586 | 1 | 50.7 | 5,039 | a combination of Illumina and 454 technologies | *stx_2a_*+; *aggr+, aatA* +, and positive for fermentation of sorbitol, lactose  and *β*-glucuronidase.  *eae; ehxA-,* and *CefS* (Republic of  Georgia) |
| O104:H4 | 2011C-3493 | NC_018658.1 | 5,273,097 | 2 | 50.7 | 4,974 | a combination of Illumina and 454 technologies | *stx_2a_*+; *aggr+, aatA* +, and positive for fermentation of sorbitol, lactose  and *β*-glucuronidase.  *eae; ehxA-,* CTX15M-positive and *CefR* (Germany) |
| O104:H4 | LB226692 | AFOB02000001-AFOB02000356;  SRR254209 | 5,189,625 | na | 50.7 | na | Ion Torrent | stool sample from patient with hemolytic uremic syndrome (Germany) |
| O104:H4 | TY-2482 | AFOG01000001-AFOG01000451  SRX067313 | 5,178,909 | na | 50.7 | na | Ion Torrent | stool sample from patient with hemolytic uremic syndrome (Germany) |
| O104:H4 | C760-09 | SRX078303 | 4,894,831 | na | 50.9 | na | PacBio SMRT™ | Stool sample from child without diarrhea, not from a single clonal outbreak (Africa, 2008) |
| O104:H4 | C777-09 | SRX078304 | 5.207,617 | na | 50.8 | na | PacBio SMRT™ | Stool sample from child with diarrhea, not from a single clonal outbreak (Africa, 2008) |
| O104:H4 | C734-09 | SRX078308 | 5,114,139 | na | 50.7 | na | PacBio SMRT™ | Stool sample from child without diarrhea, not from a single clonal outbreak (Africa, 2008) |
| O104:H4 | C754-09 | SRX078302 | 5,239,818 | na | 51.4 | na | PacBio SMRT™ | Stool sample from child without diarrhea, not from a single clonal outbreak (Africa, 2008) |
