# Supplementary material for: Genome sequencing and comparative genomics provides insights on the evolutionary dynamics and pathogenic potential of different H-serotypes of Shiga toxin-producing Escherichia coli O104
Source: BMC Microbiol. 2015 Apr 3;15:83. doi: 10.1186/s12866-015-0413-9 (PMC4393859; doi:10.1186/s12866-015-0413-9)
Supplement: Additional file 2: Table S2. — Distribution and general information of the predicted prophages in various H types of O104 strains. [file 12866_2015_413_MOESM2_ESM.docx]

**Table S2:** Distribution and general information of the predicted prophages in various H-serotypes of O104 strains.

| SEROTYPE (strain) | REGION | REGION LENGTH | #CDS | REGION POSITION | POSSIBLE PHAGE | G+C (%) | **Shiga Toxin Gene (prophage name)** |
| --- | --- | --- | --- | --- | --- | --- | --- |
| O104:H7 (RM9387) | 1 | 17.3Kb | 22 | 1895624-1912959 | PHAGE_Pseudo_PPpW_3_NC_023006, . | 49.75% |  |
|  | 2 | Y32.8Kb | 42 | 2613951-2646798 | PHAGE_Entero_BP_4795_NC_004813, .. | 54.54% |  |
|  | 3 | 63.2Kb | 78 | 2923395-2986656 | PHAGE_Entero_BP_4795_NC_004813, ... | 52.81% | *stx2A* (PHAGE_Escher_P13374_NC_018846);  *stx2B* (PHAGE_Escher_P13374_NC_018846) |
|  | 4 | 48.1Kb | 54 | 3426939-3475100 | PHAGE_Entero_lambda_NC_001416, ...... | 49.88% |  |
|  | 5 | 14.1Kb | 18 | 4173080-4187264 | PHAGE_Entero_P4_NC_001609, ...... | 50.26% |  |
| O104:H21 (94-3024) | 1 | 49.4Kb | 46 | 1527885-1577296 | PHAGE_Salmon_SP_004_NC_021774, ... | 51.90% |  |
|  | 2 | 56.2Kb | 69 | 1771892-1828174 | PHAGE_Entero_phiP27_NC_003356, ...... | 51.82% | *stx2A* (PHAGE_Escher_P13374_NC_018846);  *stx2B* (PHAGE_Escher_P13374_NC_018846) |
|  | 3 | 30.6Kb | 38 | 2739289-2769950 | PHAGE_Entero_BP_4795_NC_004813, .... | 55.46% |  |
|  | 4 | 24.8Kb | 35 | 3525724-3550609 | PHAGE_Entero_lambda_NC_001416, .... | 47.90% |  |
| O104:H4 (2009EL-2050) | 1 | 72.9Kb | 70 | 2121801-2194700 | Enterobacteria phage mEp460 | 51.26% |  |
|  | 2 | 42.5Kb | 49 | 2340286-2382874 | PHAGE_Yersin_413C | 52.22% |  |
|  | 3 | 66.8Kb | 73 | 2515070-2581889 | PHAGE_Stx2_c_II | 49.78% |  |
|  | 4 | 47.3Kb | 62 | 2884130-2931484 | Enterobacteria phage HK630 | 51.48% |  |
|  | 5 | 62.6Kb | 74 | 3246377-3308979 | Escherichia phage TL-2011c | 50.23% | *stx2A* (PHAGE_Escher_TL_2011c);  *stx2B* (PHAGE_Escher_TL_2011c) |
|  | 6 | 58.3Kb | 65 | 3559411-3617791 | Enterobacteria_phage_lambda | 51.19% |  |
| O104:H4 (2011C-3493) | 1 | 46.3Kb | 40 | 1558077-1604475 | Enterobacteria_phage_HK620 | 45.38% |  |
|  | 2 | 70.3Kb | 70 | 2133761-2204067 | Enterobacteria phage mEp460 | 51.36% |  |
|  | 3 | 43.7Kb | 50 | 2349653-2393440 | PHAGE_Yersin_413C | 52.26% |  |
|  | 4 | 66.8Kb | 74 | 2525541-2592360 | PHAGE_Stx2_c_II | 49.78% |  |
|  | 5 | 47.3Kb | 61 | 2892626-2939977 | Enterobacteria phage HK630 | 51.49% |  |
|  | 6 | 62.8Kb | 74 | 3254121-3316983 | Escherichia phage P13374 | 50.26% | *stx2A* (PHAGE_Escher_P13374_NC_018846);  *stx2B* (PHAGE_Escher_P13374_NC_018846) |
|  | 7 | 58.3Kb | 65 | 3567415-3625788 | Enterobacteria_phage_lambda | 51.19% |  |
| O104:H4 (2009EL-2071) | 1 | 46.3Kb | 40 | 1553031-1599429 | Enterobacteria_phage_HK620 | 45.38% |  |
|  | 2 | 72.9Kb | 70 | 2129491-2202396 | Enterobacteria phage mEp460 | 51.26% |  |
|  | 3 | 42.5Kb | 49 | 2347982-2390570 | PHAGE_Yersin_413C | 52.22% |  |
|  | 4 | 66.8Kb | 74 | 2522766-2589585 | PHAGE_Stx2_c_II | 49.78% |  |
|  | 5 | 47.6Kb | 62 | 2891050-2938742 | Enterobacteria phage HK630 | 51.55% |  |
|  | 6 | 47.6Kb | 62 | 3053835-3101448 | Enterobacteria phage HK630 | 51.84% |  |
|  | 7 | 62.6Kb | 73 | 3304002-3366604 | Escherichia phage TL-2011c | 50.23% | *stx2A* (PHAGE_Escher_TL_2011c);  *stx2B* (PHAGE_Escher_TL_2011c) |
|  | 8 | 58.3Kb | 63 | 3617035-3675415 | Enterobacteria_phage_lambda | 51.18% |  |
