# Supplementary material for: Genome sequencing and comparative genomics provides insights on the evolutionary dynamics and pathogenic potential of different H-serotypes of Shiga toxin-producing Escherichia coli O104
Source: BMC Microbiol. 2015 Apr 3;15:83. doi: 10.1186/s12866-015-0413-9 (PMC4393859; doi:10.1186/s12866-015-0413-9)
Supplement: Additional file 3: Figure S1. — Proposed evolutionary model of the selC-tRNA site from various H-types of E. coli O104 strains. [file 12866_2015_413_MOESM3_ESM.pptx]

## Slide 1
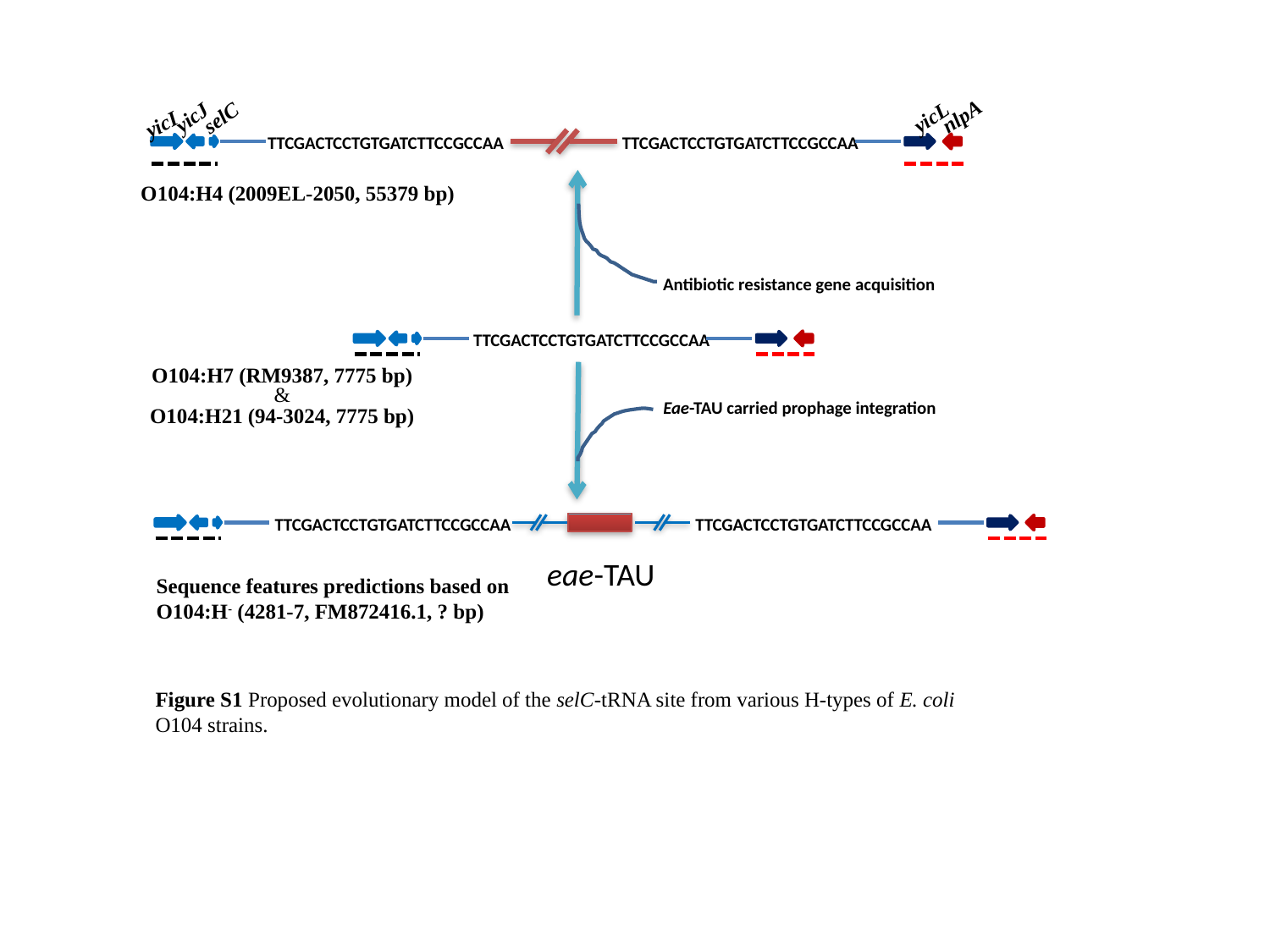

nlpA
yicJ
yicL
selC
yicI
TTCGACTCCTGTGATCTTCCGCCAA
TTCGACTCCTGTGATCTTCCGCCAA
O104:H4 (2009EL-2050, 55379 bp)
Antibiotic resistance gene acquisition
TTCGACTCCTGTGATCTTCCGCCAA
O104:H7 (RM9387, 7775 bp)
&
O104:H21 (94-3024, 7775 bp)
Eae-TAU carried prophage integration
TTCGACTCCTGTGATCTTCCGCCAA
TTCGACTCCTGTGATCTTCCGCCAA
eae-TAU
Sequence features predictions based on
O104:H- (4281-7, FM872416.1, ? bp)
Figure S1 Proposed evolutionary model of the selC-tRNA site from various H-types of E. coli
O104 strains.
